# Review of Decompression Damage of the Polymer Liner of the Type IV Hydrogen Storage Tank

**DOI:** 10.3390/polym15102258

**Published:** 2023-05-10

**Authors:** Zeping Jin, Ying Su, Hong Lv, Min Liu, Wenbo Li, Cunman Zhang

**Affiliations:** 1 School of Automotive Studies, Tongji University, Shanghai 201804, China; jinzeping@tongji.edu.cn (Z.J.); shmily_suying@163.com (Y.S.); lvhong@tongji.edu.cn (H.L.); 2 Clean Energy Automotive Engineering Center, Tongji University, Shanghai 201804, China; 3 Research Institute of State Grid Zhejiang Electric Power Co., Ltd., Hangzhou 310005, China; liumhb@126.com

**Keywords:** fuel cell electric vehicles, type IV hydrogen storage tank, liner, polymer, decompression damage

## Abstract

The type IV hydrogen storage tank with a polymer liner is a promising storage solution for fuel cell electric vehicles (FCEVs). The polymer liner reduces the weight and improves the storage density of tanks. However, hydrogen commonly permeates through the liner, especially at high pressure. If there is rapid decompression, damage may occur due to the internal hydrogen concentration, as the concentration inside creates the pressure difference. Thus, a comprehensive understanding of the decompression damage is significant for the development of a suitable liner material and the commercialization of the type IV hydrogen storage tank. This study discusses the decompression damage mechanism of the polymer liner, which includes damage characterizations and evaluations, influential factors, and damage prediction. Finally, some future research directions are proposed to further investigate and optimize tanks.

## 1. Introduction

As the lightest and the most abundant element, hydrogen has the advantages of high calorific value and zero emissions [1]. It is the ideal new energy for the 21st century [2]. Fuel cell electric vehicles (FCEVs) are one of the applications of hydrogen energy in vehicles. However, due to the low density of hydrogen at ambient temperature, realizing high hydrogen storage density is one of the critical issues in the development of FCEVs. Thus, the progress of advanced on-board hydrogen storage methods is attracting global attention [3,4].

Among the existing hydrogen storage methods, high-pressure gaseous hydrogen storage is presently the most common and mature choice for on-board hydrogen storage [5]. It stands out for its low storage energy consumption, fast charging and discharging, outstanding dynamic response, and wide temperature operating range [6]. Meanwhile, the research into and applications of high-pressure fuel gas tanks are relatively mature, especially compressed natural gas (CNG) tanks, which can be a reference for studying high-pressure hydrogen storage tanks.

**Figure 1:**
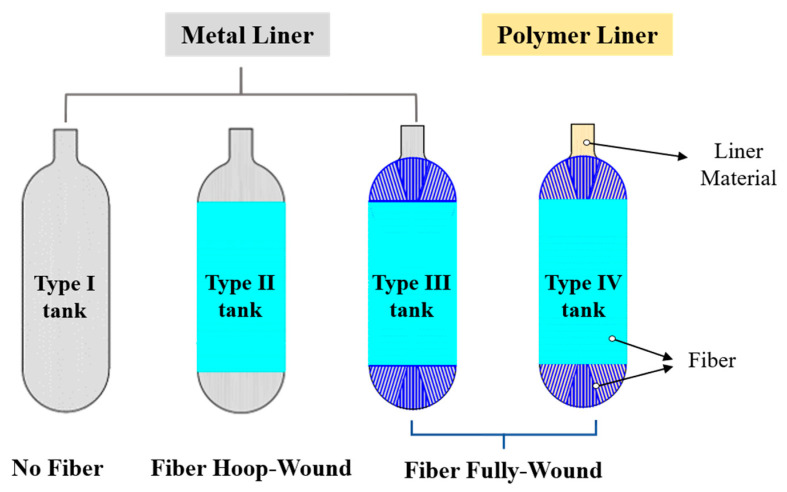
Four types of hydrogen storage tanks.

There are currently four main types of hydrogen storage tanks. Figure 1 shows the all-metal gas tank (type I), the metal-lined gas tank hoop-wound with fiber (type II), the metal-lined gas tank fully wound with fiber (type III), and the polymer-lined gas tank fully wound with fiber (type IV). These four types have achieved a significant transition to a product with a more reasonable structure, lower weight, and better economy. The type I and type II tanks with steel liners have some disadvantages, such as high weight and serious hydrogen embrittlement [7,8]. These disadvantages cannot meet the requirements of on-board hydrogen storage systems such as lightness and safety. In contrast, the type III and type IV tanks achieve the weight reduction and improve the hydrogen storage density [9,10]. Therefore, they are more widely adopted in the FCEVs industry. So far, some cylinder manufacturers and automobile manufacturers have successfully developed a variety of specifications of fiber fully wound high-pressure hydrogen storage tanks. In 2001, Quantum designed the type IV TriShieldTM cylinder, with the highest storage pressure at 70 MPa [11,12]. In 2018, Hyundai introduced a new FCEV, NEXO, with three 700-bar fuel tanks. Compared to its first FECV SUV, its storage density improved from 4.4 wt% to 5.7 wt% [13]. In Japan, following the release of MIRAI in 2014 [14], Toyota developed a new generation of MIRAI in 2020 [15]. The new storage system configured three type IV tanks with a filling pressure of 70 MPa, increasing the hydrogen capacity and driving range by about 20% and 30%. Meanwhile, its gravimetric density reached 6.0 wt%, which has achieved the goal set by DOE for 5.5 wt% by 2025 [16].

The type IV tank possesses excellent corrosion and fatigue resistance due to its polymeric liner [17]. The options for its material liner are still in the exploratory stage and include, but are not limited to, high-density polyethylene (HDPE), polyamide (PA), polyethylene glycol terephthalate (PET), and series materials of polyether [18,19]. Meanwhile, some studies are interested in polymer blends, for example, a mixture of 95 wt% low-linear-density polyethylene (LLDPE) and 5 wt% HDPE [20]. Compared to the type III tank, the type IV tank is lighter and cheaper. The complicated manufacturing process and long manufacturing cycle for metal liners make the type III tank costly. In addition, the poor fatigue performance of metals requires the type III tank to be equipped with many high-modulus carbon fibers to extend its service life, which further increases the cost. By contrast, the low price of raw materials and the simple molding process allow the type IV tank to achieve a low cost. The future development of high-pressure hydrogen storage tanks aims at a light weight, low cost, high storage density, and long service life. Thus, the market for 70 MPa type IV tanks for hydrogen-powered cars is potentially extensive.

In type IV tank applications, the structural and performance integrity of the polymer liner is of great importance and must be guaranteed. Due to the hydrogen permeation through polymers [16], the coupling between gas diffusion and mechanical properties may compromise this integrity on rapid gas decompression. Rapid gas decompression is a recognized phenomenon in polymer applications. It generally occurs in some extreme situations: system failure, component damage, periodic inspections, and aging tests. Such gas decompression may have potential for severe damage inside the plastic liner (blistering, whitening, voids, cavities, or cracks). This damage may have serious consequences, increasing the likelihood of premature failure. On the one hand, the liner’s mechanical properties will deteriorate. On the other hand, the damage will aggravate hydrogen permeation. Therefore, a thorough understanding of the mechanism of the polymer liner’s decompression damage is of great significance in improving the safety of tanks. There are few similar reviews in this field. References [18,21] covered a wide range of studies related to the application of polymer materials in a hydrogen atmosphere. However, the above-mentioned reviews did not include in-depth discussion on decompression damage.

This paper comprehensively reviews, categorizes, and summarizes the relevant literature on the decompression damage of polymer liners, analyzing the decompression damage of liner materials in terms of damage mechanisms, characterization methods, influencing factors, and prediction models, and further providing an outlook on future research directions. Section 2 describes the hydrogen behavior inside polymers during decompression. The characterization methods and quantitative evaluations of damage morphology in existing research are summarized in Section 3. Section 4 lists the factors affecting the internal damage of polymers, including the material level and operation condition. Section 5 describes the predictive models and risk assessments that may be utilized for decompression damage. Some future research directions are proposed in Section 6. 

This paper provides a more comprehensive understanding of the decompression damage in the polymer liner of type IV hydrogen storage tanks and provides guidance for future standard test methods, liner design, hydrogen storage system control strategies, and predictive tool development.

## 2. Hydrogen Behavior through Polymeric Materials during Decompression

The decompression process involves gas transport: under high-pressure hydrogen soaking, gas molecules permeate through the polymer and then gradually occupy it; when gas decompression occurs, the gas molecules inside escape to reach a new equilibrium.

### 2.1. Gas Permeation through Polymers

Polymeric materials are well known to be permeable to gases [22]. As the smallest molecule, hydrogen gas can dissolve in and diffuse through the polymer. A solution-diffusion mechanism can explain the gas permeation behavior through a polymeric material. The permeation behavior of hydrogen molecules in polymeric materials involves hydrogen dissolution at the surface of the materials and hydrogen diffusion inside the material [23]. Flaconneche et al. [24] divided the transport process into the three consecutive events listed below:

1. The polymer absorbs the gas on the high-pressure side.
2. The gas diffuses inside the polymer matrix.
3. The polymer desorbs the gas on the low-pressure side.

**Figure 2:**
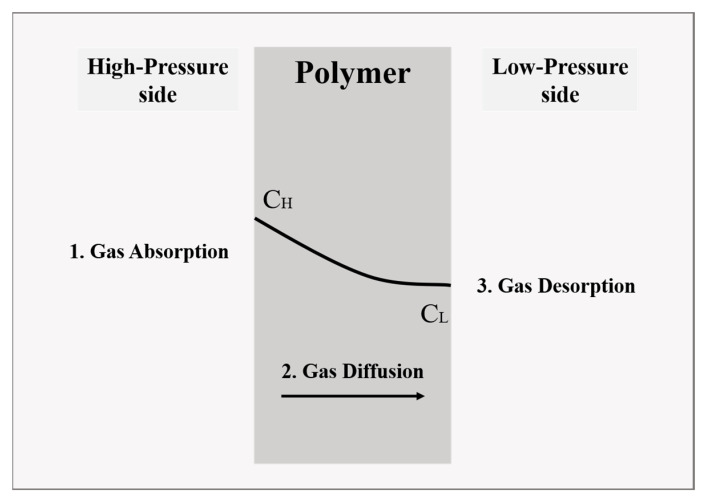
The schematic illustration of gas transport through polymers.

This process is described in Figure 2, where the black solid curve represents the gas concentration gradient, and *C_H_* and *C_L_* represent the gas concentration on the high-pressure and low-pressure sides, respectively.

Previous literature has highlighted three properties to characterize the gas permeation process: solubility, diffusivity, and permeability. The first is a thermodynamic property describing the amount of gas that the material can absorb. The second is a kinetic property that describes the speed at which gas molecules move from one side to the other. Finally, *P* represents the ability of the gas to pass through the polymer. The relationship of coefficients corresponding to three properties (diffusivity coefficient *D*, solubility coefficient *S*, and permeability coefficient *P*) are expressed as Equation (1).

(1) P=S×D

Gas diffusion is the dominant parameter of permeation since it is very slow [22]. Diffusion was proved to exist only in the amorphous regions but to be completely restricted in the crystalline regions [25]. Gas molecules can move from one position to another by chain segment movement in the free volume, thus allowing gas diffusion inside the polymer [26,27].

### 2.2. Formation of Decompression Damage

The amount of hydrogen permeating the liner is tiny, which seems negligible during the service period of the tank. However, the negative consequence of permeation is usually fatal: damage may occur inside the liner under rapid decompression, such as cracks, blistering, whiting, cavities, and voids. This phenomenon exists in both elastomers and thermoplastics, but elastomers are generally more severe than thermoplastics [28]. Scholars have studied this phenomenon for years [29,30]. 

**Figure 3:**
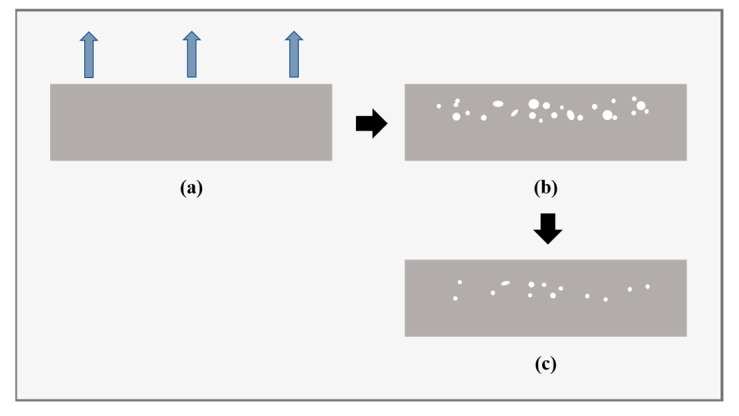
Schematic representation of the decompression damage inside the liner: (**a**) at the beginning of gas decompression, (**b**) at the end of gas decompression, and (**c**) several hours after gas decompression.

Generally, internal damage occurs due to the huge pressure difference between the inside and outside of the liner. Rapidly decreasing ambient pressure coupled with slow gas diffusion results in gas supersaturation. In the gas-release process, the original equilibrium created under high pressure is out of balance. The penetrated hydrogen cannot diffuse out of the material quickly since it is trapped inside, contributing to the high gas concentration. According to Henry’s law, the trapped hydrogen generates high pressure inside the polymer. The pressure difference between the inside and outside of the polymer then rapidly increases. As a result, stress concentration is generated around the microscopic pores or existing defects or at the interface between the polymer and additives inside the liner. When the stress or strain exceeds the limit, irreversible damage occurs, which results from a combination and interaction of thermal, diffusion, and mechanics effects. It has been reported that the damage partially disappears several hours after the end of gas decompression [31], as illustrated in Figure 3. This phenomenon indicates that the damage may have two forms: reversible and irreversible damage. In the literature, the damage mainly occurs in the amorphous region or the less crystalline areas in the semi-crystalline polymer [32,33] and the areas of low structural strength in rubbers [34,35].

It is confirmed that the cracks result from plastic deformation and remain after hydrogen is desorbed entirely [32]. As shown in Table 1, the appearance of damage may harm some mechanical properties of materials [28,36]: the loss in stiffness, the drop in yield stress, and the reduction in rupture stress. Nevertheless, the damaged material became more resilient as the elongation at the break and the impact energy improved [28]. The internal damage may aggravate gas permeation as the damage sites shorten the diffusion paths to promote gas transport [36]. More seriously, liner collapse, a permanent deformation, may occur. Research has shown that the ratio of material yield stress to Young’s modulus ratio determined the critical pressure for liner collapse [37]. Thus, as the internal damage propagates during compression–decompression cycles, it could lead to catastrophic and expensive structural failure. 

## 3. Characterization Methods and Quantitative Evaluations for Internal Damage Morphology in Polymers

Damage morphology often occurs inside the material, increasing the difficulty of characterization. The characterization method is used to determine whether there is damage in the material and to estimate the extent of the damage. A reliable damage characterization method plays a significant role in the investigation of damage mechanisms and is a guide for engineering diagnosis and analysis. Naebe et al. [38] summarized the most widely used non-destruction testing methods for cracks in polymers and composites, including the types of defects observed, merits, and limitations. This section summarizes the existing characterization methods and quantitative evaluation techniques for damage detection in polymers.

### 3.1. Characterization Methods for Internal Damage of Polymers

Internal damage can be observed macroscopically and microscopically. The characterization methods mainly include light microscope/camera observation, electron microscopy observation, atomic force microscope observation, computed tomography observation, and in-situ observation. 

#### 3.1.1. Light Microscope/Camera Observation

**Figure 4:**
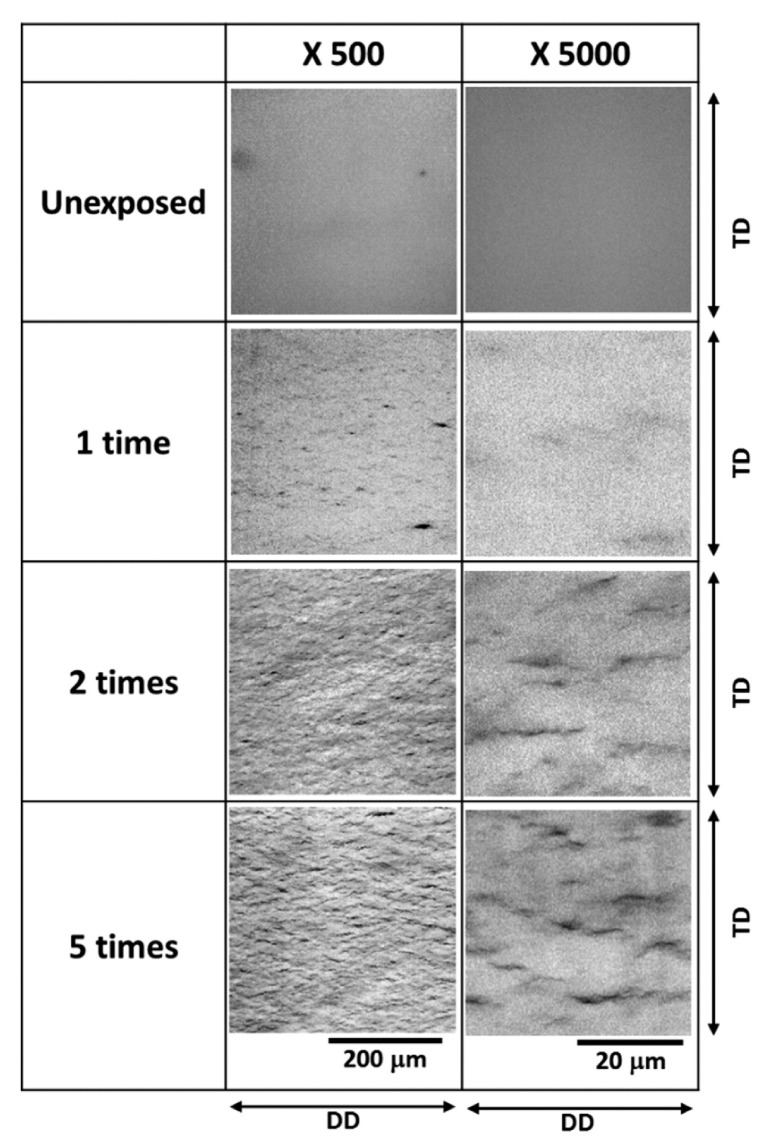
The cross-section of a thin slice cut from the center of the sample under a microscope [41]. TD refers to thickness direction and DD refers to diameter direction.

Microscope or camera observation is a common and straightforward way to detect damage [39,40]. Kaga et al. [35] used an optical microscope to observe the blistering behavior of the O-ring specimen from the direction of the pressurized side through a glass viewport. Hiroaki et al. [41] used an optical microscope equipped with a high-resolution lens to view a 0.2–0.3 mm thick slice cut from the center of the sample along the thickness direction. They focused on about 0.1 mm inside the slice rather than on the surface. Thus, the obtained pictures (Figure 4) reflected the size, density, and direction of cracks. Baldwin [42] also took the thin cross-sections from the center of the samples and inspected the minor fractures under a stereo microscope with the assistance of polarized light.

#### 3.1.2. Electron Microscopy Observation

Compared with light microscopy, electron microscopy is an effective method to provide higher-resolution images that are difficult to attain. Transmission Electron Microscopy (TEM) and Scanning Electron Microscopy (SEM) are the two most common types of electron microscopy. TEM sends a beam of electrons through the sample, producing detailed 2D projection images of the internal structure, including morphology, composition, and crystal structure. The sample should be extremely thin to allow the electron beam to pass through. The cryo-ultramicrotomy technology is the most basic method of sample preparation for TEM [43]. Gerland et al. [33] discussed the early stages of cavitation in poly(vinylidene fluoride) (PVDF) on ultrathin specimens approximately 60–80 nm thick at the nanometric scale. Nanobubbles gathering into alignments were observed. SEM utilizes a focused electron beam to scan the sample for surface images in 3D. It has no limit to the thickness of the sample. Dewimille et al. [28] broke a decompression-damaged PVDF by cold fracturing and then employed SEM to exhibit its visible microscopic damage after the blistering test. EDS (energy-dispersive X-ray spectrometry) and EDX (energy-dispersive X-ray spectrometry) are supplementary means for SEM to obtain the element images. Yamabe et al. [39] utilized SEM coupled with EDS to observe the fracture surfaces of O-rings. They supposed that the facet on the fracture surface was a trace of bubbles. Sulfur and zinc oxide were considered to be the reason for the defect in the rubber [40].

#### 3.1.3. Atomic Force Microscope (AFM)

AFM is a scanning probe microscope with nanometer resolution, whose role is to obtain the 3D surface information of samples, including surface morphology and roughness. Researchers utilized AFM to study the surface morphology of damaged polymers [34,35]. They compared the height images of exposed and unexposed samples to study the origin and development of nanoscale fractures through the average number, length, depth, and width of line-like structures. However, the observation area is relatively small and requires multi-region testing to ensure universal results.

#### 3.1.4. Computed Tomography (CT)—3D Technique

CT, a 3D imaging technique, has been introduced to describe more detailed and abundant information inside polymers. This technique is based on the attenuation and absorption of radiation by the detected object. It uses X-rays to take successive cross-sectional images inside an object, and then a computer reconstructs a 3D image of the internal structure from the set of radiographs. CT provides a 3D image set that reveals the distribution, nature, and shape of the damage [44,45,46]. Owing to the diversity in density, the cracks and polymer matrix reflect the different gray values on CT images. The micro-CT images helped Kulkarni et al. [47] infer the damage sensitivity of three types of EPDM (pure, plasticized, and filled with carbon black fillers) and validate their simulation results qualitatively. In the literature [32], researchers obtained images of LDPE at different magnifications. They found that cracks often appeared parallel to the surface and in the center of samples along the thickness. At the same time, several large cracks were observed, along with a large number of small cracks at high magnification.

#### 3.1.5. In-Situ Observation

In-situ observation is employed to capture more information about the increase in damage. It is necessary to monitor the evolution of damage in samples throughout the test period without moving samples and disrupting the continuity of the experiment. In addition, the fixed position of the sample throughout the observation period makes it easy to locate the damage and analyze its growth. In the previous research on rubber and elastomers (PDMS and EPDM), the authors conducted a time-resolved follow-up with an optical microscope or camera to study the growth behavior of internal damage and obtain information such as cavity initiation time and the number, size, and distribution of cavities [31,48]. To better understand the decompression failure mechanisms in rubber exposed to high-pressure hydrogen, Sylvie et al. [49] first utilized in-situ X-ray CT to obtain time-resolved 3D images. Nevertheless, the existing problem for this in-situ X-ray CT is to better coordinate the contradiction between the acquired image data quality and the damage evolution’s kinetics. Moreover, the apparatus complexity of the in-situ observation increases extensively more than that of the ex-situ observation.

### 3.2. Quantitative Evaluations for Internal Damage of Polymers

Generally, the quantitative evaluation aims to quantify the damage and provide more compelling data to support damage mechanism studies.

#### 3.2.1. Optical Method

One optical method applied light scattering theory on transparent and semitransparent materials [32,41]. The existence of internal damage scatters incident light, leading to a decline in transmitted light intensity. Therefore, the percentage of light extinction determined the extent of internal damage. The transmitted light image acquisition systems mainly consist of an LED light source, tested samples, and a camera (from bottom to top). Since polyethylene is optically inhomogeneous, a relative extinction ratio ($\beta_{r}$) was introduced to avoid the influence brought by the original scattering of the transmitted light. Equation (2) indicates the relationship between $\beta_{r}$, $B_{BF}$ (the brightness of the unexposed sample), and $B_{AF}$ (the brightness of the exposed sample).

(2) $$\beta_{r}=1-\frac{B_{AF}}{B_{BF}}$$

Considering the influence of crystallinity on the transparency of samples, Fujiwara et al. [32] measured crystallinity before and after the decompression. Although the crystallinity of polyethylene (PE) increased under high-pressure hydrogen, it did not change after decompression, which meant that its influence on the light transmission could be negligible in this case. Equation (2), therefore, succeeds in evaluating decompression damage but is limited by the transparency of samples.

Another optical method assessed the degree of damage with the assistance of dye to distinguish cracks from the polymer matrix. Briscode et al. [50] observed thin 1 mm cross-sections cut from the specimens. To highlight the cracks, they soaked the sections in black ink and then employed an image analyzer to calculate three damage indexes: the proportion of crack area (%), the number of cracks, and the mean length of cracks (mm).

#### 3.2.2. Mechanical Method

Sometimes, polymers are not transparent as pure materials or filled with fillers. Therefore, the optical method mentioned above may not be applicable to these opaque samples. Some studies adopted some mechanical methods to quantify the overall damage inside polymers. Combined with the data from the optical method, Briscode et al. [50] quantified the degree of the decompression damage using three mechanical property analyses: simple diametric compression, cyclic diametric compression, and sinusoidal tensile deformation. Lorge et al. [36] quantified the decompression damage produced in poly(vinylidene fluoride) (PVDF) based on continuum damage mechanics (CDM). The basic idea of this method was that the occurrence of damage will lead to a reduction in mechanical properties, such as Young’s modulus. Equation (3) provides the relationship between the damage variable ($\lambda_{D}$) and Young’s modulus (E for undamaged samples and $E^{\prime }$ for damaged samples) under the assumption of effective stress. Thus, the loss of Young’s modulus can measure and identify the overall extent of internal damage.

(3) $$\lambda_{D}=1-\frac{E^{\prime }}{E}$$

#### 3.2.3. Physical Property Method

Density [28,41] (or volume [39,44,51]) measurement is reported in some studies for damage quantification. Dewimille et al. [28] managed to visualize micro damage using a 2D X-ray scanner as the density gradient (areal density) on the cross-section of PVDF after rapid decompression. The results show a sharp decrease in density from the edge to the middle of the sample. As the linear relationship between density and the mean grey level value in CT images [45], the density of materials can be expressed as shown in Equation (4).

(4) $$\rho =\rho_{u}(\frac{MGL-MGL_{air}}{MGL_{u}-MGL_{air}})$$

where ρ and MGL refer to the density and mean grey level value of the damage zone, and subscripts u and air, respectively, refer to unaltered material and air.

Unfortunately, Hiroaki et al. [41] failed to identify internal damage using the density of samples. According to the idea that an increase in the degree of internal damage leads to an increase in the volume of samples, they plotted the expansion ratio of HDPE (the density ratio of the undamaged HDPE sample to the damaged HDPE sample) as a function of $\bar{\beta_{r}}$ (mean $\beta_{r}$ in the center of the specimen). The result was that the ratio was kept close to 1.00 at all exposure times. In this case, the volume measurement seemed unavailable for the assessment of defects. There are two possible explanations for this result. One is that the compression effect of high pressure counteracted the expansion effect of rapid decompression. Another reason could be that the sample (Φ 13 mm × 2 mm) was too small, and the expansion ratio was susceptible to measurement errors.

#### 3.2.4. Acoustic Emission (AE) Method

The AE method is based on the elastic waves generated when solid materials release stored energy during the fracture of the chain molecules. It has been applied to effectively monitor failure behaviors in metal [52], concrete [53], and composite materials [54]. Yuan et al. [55] applied the AE method to the tensile test of HDPE liner for type IV tanks, and the method proved to be feasible. They found that AE signals could be used to distinguish plastic deformation and fracture. Meanwhile, Yamabe et al. [56] demonstrated its feasibility for the detection of internal damage growth in sealing rubber material. They conducted AE detection in air and measured the AE event count every 5 min. The relationship between the AE event count (N) and amplitude (V) is obtained as follows:

(5) $$N=AV^{-m}$$

In this equation, the *A* value and the *m* value are constants. The *m* value is connected with the extent of the internal fracture; the more serious the fracture is, the smaller the value is [57]. Yamabe et al.’s research results indicated that the m value decreased from 3.8 to 2.1 when the exposure pressure increased from 0.7 to 10 MPa (no cracks under 0.7 MPa and cracking under 10 MPa) [56]. It is noted that the AE method is only suitable for the detection of dynamic defects instead of static defects, which significantly increases its limitations.

## 4. Factors Affecting the Internal Decompression Damage of Polymers

The safety and reliability of liners are crucial factors in accelerating the development of type IV tanks. The occurrence of decompression damage inside a liner will lead to an increased possibility of risk. It is therefore essential to determine its influencing factors as a safety reference for product design and use. Generally, the factors influencing decompression damage can be categorized into material level and operation condition.

### 4.1. Factors from the Material Level

The material itself has a major impact on decompression damage. Factors at the material level are summarized as follows.

#### 4.1.1. Properties of Polymers

As mentioned in the previous section, internal damage mainly depends on both mechanical and gas-transport properties [58]. Some works have shown that different types of polymers responded differently to gas decompression: the damage morphology of HDPE was more severe than that of PA [30,42,59]. This can be partially explained by the fact that HDPE has a lower yield stress than PA. Indeed, hydrogen bonds, a particular type of intermolecular force, allow PA to achieve higher strength and module than HDPE.

**Figure 5:**
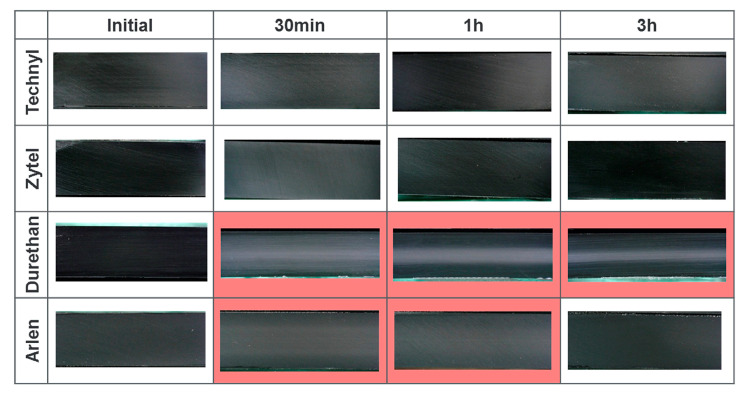
Cross section of PA liners before and after hydrogen decompression [30].

Even with the same polymer matrix, there are differences in the properties of different brands. As shown in Figure 5, researchers conducted experiments on different brands of PA and found that some samples showed varying degrees of blistering, and some even experienced no blistering [30]. It was inferred that mass diffusivity varied from one brand of PA to another. The higher diffusivity would lower the risk of cavitation as gas could escape from the polymer faster [35,60].

Research showed that more severe destruction occurred with smaller crystallinity in materials [32]. Kane calculated the flux of hydrogen through the material. The calculation results demonstrated that the flux of hydrogen increased as the crystallinity decreased, confirming that crystallinity was an indicator to assess the gas barrier performance of materials [26]. Since the gas only dissolves in the amorphous region, the high crystallinity hinders gas permeation through polymers [61,62]. The more gas accumulates in the polymer, the more stress it tends to generate by decompression.

#### 4.1.2. Defects Inside Polymers

Some defects may inevitably form inside the polymers, contributing to the stress concentration during gas decompression. The gas pressure in pre-existing defects may lead to the initiation and propagation of cracks. Prachumchon located spherical flaws with various radii at different locations to simulate the development of cracks inside HDPE [63]. The flaw’s location determined the maximum internal pressure, deciding whether the damage evolved. Gas bubbles were considered to be micron-size defects as the starting point of blister initiation in rubber structures [35].

#### 4.1.3. Additives

Additives are a typical means to improve the performance of polymers in the actual manufacturing process. Additives mainly include plasticizers, fillers, compatibilizers, crosslinking agents, and nucleating agents. The addition of nucleating agents changed the mechanics and impermeability of materials [64,65]. The influence of plasticizers appeared to aggravate the degree of damage, change the nature of damage [47], and promote gas permeation at the same time [23,62]. The fillers such as silica [58], graphene [66], clays [67], and silicate nanofillers [68] reduced the chance of damage because they allowed a decrease in hydrogen permeation and an increase in mechanical properties.

#### 4.1.4. Processing Technology

**Figure 6:**
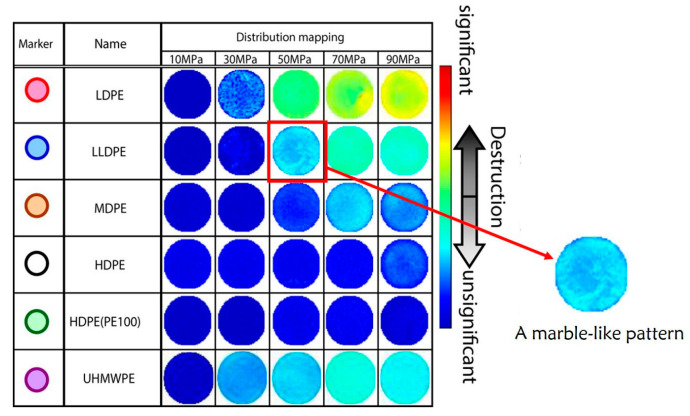
Destructive quantitation map of different types of PE under 10, 30, 50, 70, and 90 MPa [32].

The processing technology is also a crucial factor for decompression damage. As shown in Figure 6, a marble-like pattern was observed in LLDPE at 50 MPa. This particular phenomenon could be explained by the effect of resin flow during molding [32]. The existence of inhomogeneous structures has been reported in rubbers [69]. It appeared as a nanoscale line-like structure with low strength and was the origin of nanoscale fractures [34,35]. Furthermore, molding methods (injection, extrusion, blowing, and rotational molding) [70,71,72] and conditions (temperature, pressure, and cooling mode) [65] have been reported to influence the gas permeation and mechanical properties, both of which are concerned with decompression damage. Given that high processing temperature promotes the degradation of the polymer structure, it is essential to choose the optimal temperature to promote the performance of polymers.

#### 4.1.5. Thickness of Samples

**Figure 7:**
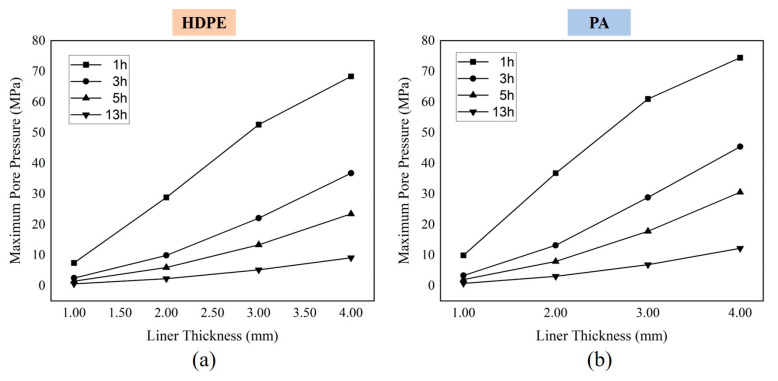
Predictive maximum pore pressure for HDPE (**a**) and PA (**b**) liners as a function of liner thickness and decompression rate (1 h, 3 h, 5 h, and 13 h) at 25 °C. (The data are derived from Reference [30]).

In principle, the thickness will not significantly influence the diffusivity, solubility, and permeability of materials [62], but the failure phenomena of different thicknesses differ under the same conditions. The most direct reason is that the thickness of the test piece determines the diffusion path of the gas in it. Previous research on the failure of polymers exposed to high-pressure gas has shown that there was little or no damage on the edge of the sample [41,73]. This is because the depressurized gas may desorb out of the material more easily in a very short time over a short distance. Similarly, Yersak et al. [30] developed the model as a function of liner thickness for both HDPE and PA liners. As shown in Figure 7, the results showed the maximum pore pressure inside the polymer with the increase in the sample thickness. Under the same operation conditions, the thinner the sample was, the lower the maximum pore pressure generated, which meant less possibility of blistering. Melnichuk et al. [74,75] also mentioned that pore pressure increased with the increment of thickness. Thus, the geometry of the test sample has an impact on the stress state in the polymers. Reducing the thickness to improve the damage resistance of liners seems better. Nevertheless, a balance is needed between a thin liner to prevent blistering and a thick liner to provide fundamental support strength for carbon fiber winding.

### 4.2. Operation Conditions Factors

Operation conditions are another factor influencing decompression damage. They mainly include the temperature, exposure pressure, decompression rate, and charge/discharge cycle.

#### 4.2.1. Temperature

**Figure 8:**
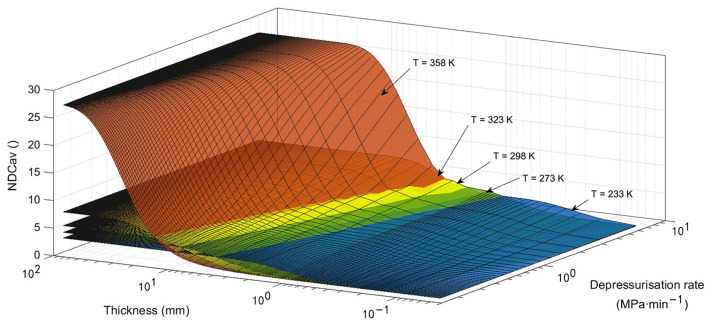
Cavitation risk at different temperatures: 233 K, 273 K, 298 K, 323 K, and 358 K [75].

At present, the application temperatures of on-board hydrogen storage tanks range from 233 K (−40 °C) to 358 K (85 °C) [5,76]. The tank suffers temperature variations as the service conditions change, such as changes in ambient temperature and the charging and discharging of hydrogen [77]. The temperature history during gas discharge is complex. There exist two effects in competition during gas decompression [78]. One is the depressurization effect: the decreased pressure leads to a decrease in the temperature of the gas and the liner material. Another is the heat transfer effect: the heat of the environment warms up the liner (when emptying, the liner’s temperature will drop below the ambient temperature). The properties of polymers are sensitive to temperature. Therefore, exploring the effect of temperature on the decompression failure behavior of liner materials is significant. To date, a few studies have reported the effect of temperature on the depressurization damage of liner materials applied to type IV tanks. As mentioned above, the material’s mechanical properties and hydrogen diffusivity play a significant role in decompression damage. In the early research on gas permeation in polymers, scholars summarized the temperature dependence of hydrogen permeability, diffusion, and solubility coefficients. They pointed out how these coefficients change with temperature, obeying Arrhenius’s laws [79,80]. At the same time, note that the material’s mechanical properties are sensitive to temperature changes. An increased temperature will lead the Young’s modulus and yield stress of PA6 [81], PA11 [82,83], and HDPE [84] to decrease, making it easier for the stress generated by the gas inside the sample to reach the critical value for material failure. Maximiliano et al. [75] fitted the hydrogen diffusivity coefficient curves and material’s yield strength of HDPE as a temperature function with literature data. They showed opposite trends with the elevation of temperature. Figure 8 shows the effect of temperature change on cavitation risk for HDPE.

#### 4.2.2. Exposure Pressure

The exposure pressure affects the internal pressure of the gas-saturated specimen and further determines the pressure difference between the internal and external when the gas is released. High pressure can create complex stress conditions. The higher the exposure pressure is, the greater the pressure difference generates during the decompression process, and the more likely it is that the internal stress will exceed the mechanical limit of the material. According to the destruction map in Figure 6, the increase in pressure enhanced the extent of the destruction [32]. Based on the numerical model, Maximiliano et al. [74] simulated the pore pressure for HDPE as a function of saturation pressure varying from 10 to 87.5 MPa and found that higher saturation pressure resulted in higher pore pressure. Baldwin [42] conducted sufficient tests on HDPE and PA samples and concluded that 35 MPa was a dividing point above which decompression damage occurred.

Meanwhile, the effect of pressure on liner properties is reflected in the permeability resistance. It is reported that the diffusivity coefficients are different in the compression and the decompression processes. Using the parameter estimation method, Prachumchon et al. [63] found that the diffusion coefficient in HDPE subjected to high pressure tended to be lower. This is because the sample shrinks under high pressure, reducing the available free volume inside the polymer matrix [32,85]. After the pressure is removed, the polymers expand as the internal free volume increases, making it easier for the gas to diffuse. Furthermore, it is worth considering whether the mechanical properties of polymers will be altered in a high-pressure hydrogen environment. Sylvie et al. [81] found no significant difference in the tensile properties of PE at the lower hydrogen pressures of 3 MPa and 10 MPa. Later, Alvine et al. [86] conducted in-situ tensile tests on HDPE in the context of hydrogen with exposure pressures of 28 MPa, 31 MPa, and 35 MPa. They observed a clear downward trend in tensile strength with increasing pressure. However, the working pressure of type IV hydrogen storage tanks is much higher than these values, such as 70 MPa. Whether and how much the mechanical properties of the polymer will change under higher pressure need further study.

#### 4.2.3. Decompression Rate

**Figure 9:**
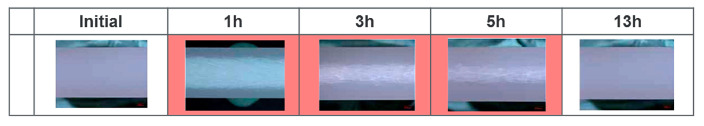
Cross section of HDPE liner before and after hydrogen decompression [30].

The decompression rate refers to the amount of external high-pressure gas released per unit of time, generally in MPa/min. The rate may determine the gas concentration gradient in polymers after decompression, thus affecting the stress value and stress distribution in polymers. In the studies of decompression damage in elastomers, the damage becomes more severe as the rate increases [87,88,89]. For type IV tank liners, Yersak et al. [30] compared the cross sections of HDPE and PA after decompression for 1, 3, 5, and, 13 h. In Figure 5 and Figure 9, the white-colored area in the center of the cross sections is the decompression-induced blistering. It is clear that the area and the density of blistering gradually decreased as the decompression time increased. These results suggested that the longer the decompression time was (the lower the decompression rate was), the less damage occurred. Consistently, the simulation results in Figure 7 showed that the pore pressure became severe as the decompression time shortened. Additionally, the function of the decompression rate on polyvinylidene fluoride exposed to carbon dioxide was studied by Baudet et al. [73]: the density of cavities grew as the decompression rate increased, and the center of the sample suffered the most intensive cavity. 

#### 4.2.4. Charge/Discharge Cycle

**Figure 10:**
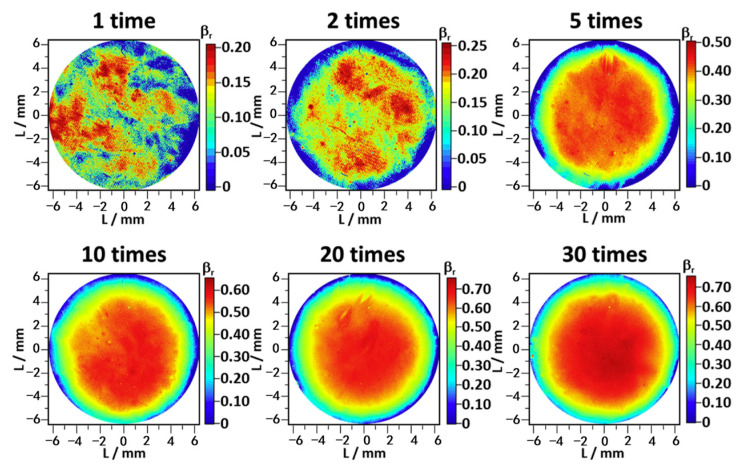
$\beta_{r}$ Map for 1, 2, 5, 10, 20, and 30 times of hydrogen exposure [41].

Hydrogen storage tanks will inevitably be exposed to frequent hydrogen charge and discharge during service. All components related to polymers in type IV hydrogen storage tanks affected by hydrogen cycles will age to some extent. In a final report [42], the author conducted 12 min cycles four times under 64.8 MPa on injection-molded HDPE. It was observed that with the increase in cycles, small cracks began to form inside the HDPE, which continued to grow and connect, eventually forming a delamination surface. Hiroaki et al. [41] skillfully used the optical method to study the effect of hydrogen exposure repetition on the internal damage of HDPE. The disk-shaped sample was placed in a hydrogen atmosphere at 30 °C and 90 MPa under a decompression rate of 360 MPa/min. Judging from the cross-sections (Figure 4) and transmittance maps (Figure 10) of the damaged HDPE, there was a significant positive correlation between the density of internal damage and the exposure number. Lorge et al. [36] have reported that the calculated value of Young’s modulus of PVDF decreased when the number of cycles increased. According to Equation (3), the more cycles the sample suffered, the higher the $\lambda_{D}$ value was. A similar phenomenon occurred in rubber when subjected to pressure cycles [48]. Yamabe et al. [39] tested O-rings with exposure cycles ranging from 3 to 300 under a hydrogen atmosphere. The results suggested that cracks of outside and inside surfaces grew with the cycles. Meanwhile, they explored the effect of the cycle pattern (at different frequencies), and the results showed that the crack damage of O-rings at 1 min/cycle was less than that at 160 min/cycle. This was because at a low pressure level, the cracks grew over time [90].

## 5. Decompression Damage Predictive Models and Assessments for Polymers

A damage-predictive model is a practical tool for polymer liners to pre-select materials and design schemes. The number of experiments required can be scaled down to reduce the time and economic cost of developing hydrogen storage tanks. The decompression damage predictions and assessments for rubbers and plastics are discussed in this section.

### 5.1. Decompression Damage Predictive Models for Polymers

The predictive model provides a clear view of the stress state and damage growth inside the sample. The following two prediction models are for sudden fracture and blistering, respectively.

#### 5.1.1. Damage Predictive Model for Sudden Fracture

Kulkarni et al. [47] simulated the stress distribution and damage evolution of three variants of EPDM, which revealed the sudden fracture and nonlinear nature. By comparing data obtained from uniaxial tensile test with fitted hyper-elastic model parameters, the Ogden model with three parameters was chosen for further analysis. According to the sudden fracture of EPDM, the authors chose the maximum principal strain failure theory as the damage model. This theory suggested that the fracture generates when the maximum principal strain $\varepsilon_{\max}$ reaches the strain at the fracture point $\varepsilon_{f}$ in the tensile test, as illustrated in Equation (6). According to the diffusion analysis, the time scale of diffusion was larger than that of damage propagation, so the hydrogen concentration was thus assumed to be constant in this study.

(6) $$\varepsilon_{\max}\geq \varepsilon_{f}$$

#### 5.1.2. Damage Predictive Model for Blistering

Based on Fick’s law, Henry’s law, and the simple material yield criterion derived from continuum mechanics, Yersak et al. [30] developed a simple blistering model for plastic liners. They simulated the effects of decompression rate and liner thickness on the blistering of HDPE and PA liners. The pre-existing pores were assumed, although no apparent pores were observed in plastic liners under the microscope. The pore pressure ($P_{pore}$) was calculated using Henry’s law in Equation (7).

(7) $$P_{pore}=\frac{c}{S}-P_{ext}$$

where c ($kg/m^{3}$), S ($kg/(Pa\cdot m^{3})$), and $P_{ext}$ (MPa) represent gas concentration, solubility, and applied external pressure, respectively. A description of the hydrogen concentration evolution at a constant decompression rate is given by Equation (8) as a function of time and the 1D direction *y* of diffusion. $C_{0}$, $C_{f}$, and D respectively, represent the initial concentration, final concentration, and diffusivity; $t_{deso}$ represents decompression time; and l represents the half thickness of the liner.

(8) $$c(y,t)=C_{0}-\frac{\left(C_{0}-C_{f}\right)}{t_{deso}}\left\{t+\frac{\left(y^{2}-l^{2}\right)}{2D}+\frac{16l^{2}}{\pi^{3}D}\sum_{n=0}^{\infty }\frac{{\left(-1\right)}^{n}}{{\left(2n+1\right)}^{3}}\exp\left[-{\left(2n+1\right)}^{2}\pi^{2}\frac{Dt}{4l^{2}}\right]\cos\left[\left(2n+1\right)\frac{\pi y}{2l}\right]\right\}$$

Equation (9) describes the yield failure criterion: when the pore pressure exceeds the critical yield pressure ($P_{y}$), plastic yielding occurs around the pore, with macroscopic manifestations of whitening and blistering.

(9) $$P_{pore}>P_{y}$$

**Figure 11:**
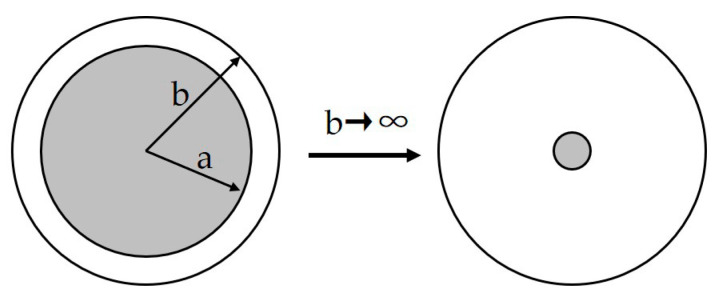
Yield criterion for embedded pores of thick-shelled pressure vessels derived from continuum mechanics.

The yield pressure $P_{y}$ is illustrated by Equation (10), which is derived from continuum mechanics. It considers the yield of the inner wall of the thick-shelled cylinder.

(10) $$P_{y}=\frac{2}{3}\sigma_{y}(1-\frac{a^{3}}{b^{3}})$$

where $\sigma_{y}$ represents the yield stress of liner materials (MPa), and *a* (m) and *b* (m) represent the inner and outer radii of the thick shell represented in Figure 11. Due to tiny pores inside the material (b≫a), Equation (10) provides a further simplified expression.

(11) $$P_{y}=\frac{2}{3}\sigma_{y}$$

This model successfully predicted the blistering phenomenon of some liner materials qualitatively. However, the predictive results of some PA brands were inaccurate. This was very likely because the hydrogen diffusivity of PA varies from brand to brand. Therefore, the diffusivity determination for each liner material helps to improve the agreement between the model and the experiment. 

The hydrogen loading pattern of this experiment and simulation is that both sides of the sample are in a hydrogen environment. In the actual situation, only one side of the liner is exposed to the high-pressure hydrogen, and the other side is exposed to the atmosphere. The gas concentration is significantly high on one side of the polymer and low on the other. The different liner–hydrogen contact patterns will therefore show the different internal damage distribution.

### 5.2. Decompression Damage Assessments for Polymers

The decompression damage assessment is used to estimate the occurrence of decompression damage for a given set of parameters.

#### 5.2.1. Assessment of Blistering Initiation Limit Pressure

The stress-instability method was proposed to estimate the blister initiation limit pressure [91]. Koga et al. [35] approximated the constitutive relationship of rubber with a neo-Hookean solid. The internal pressure of the blister ($P_{in}$) is expressed by Equation (12) based on the finite strain theory of elasticity.

(12) $$P_{in}=\frac{E}{6}(5-\frac{4}{\lambda_{a}}-\frac{1}{{\lambda_{a}}^{4}})$$

where $\lambda_{a}$ is the tangential stretch ratio at the cavity surface, and *E* is Young’s modulus. When the cavity reaches the blister initiation limit, the $\lambda_{a}$ value tends to infinity; the internal limit pressure of the blister, $P_{in,F}$, is obtained by Equation (13), whereas it is not enough to judge $P_{in,F}$ using only mechanical characteristics. The $P_{in,F}$ values of Ethylene Propylene Diene (EPDM) and Vinyl Methyl polysiloxane (VMQ) were lower than the exposure pressure in this study. However, VMQ was resistant to blistering, while EPDM was susceptible to blistering. One possible reason for this is the difference in diffusion between these two materials: the high gas diffusivity promotes gas desorption before blister formation. Thus, the assessment of $P_{in,F}$ should consider gas diffusion for high accuracy.

(13) $$P_{in,F}≅\frac{5E}{6}$$

#### 5.2.2. Non-Dimensional Assessment of Decompression Damage

According to the experimental results in the bibliography for elastomer and thermoplastics, Maximiliano et al. [60] finally proposed a generic method for cavitation failure assessment, a dimensionless assessment, which almost matched the references cited. Based on Thomas A’s model mentioned above, they introduced several dimensionless parameters: $T_{deso}$ (the non-dimensional parameter of the diffusion model in Reference [92]),$M_{y}$ (the mechanical work considering yield limit), and NDCav (the non-dimensional cavitation parameter).

(14) $$T_{deso}=\frac{D\times t_{deso}}{l^{2}}$$

(15) $$M_{y}=\frac{P_{sat}-P_{\min}}{P_{y}}$$

(16) $$NDCav=\frac{P_{pore}}{P_{y}}$$

where D, $t_{deso}$, and l are the diffusion coefficient, the decompression time, and the half thickness of the liner, and $P_{sat}$, $P_{\min}$, $P_{y}$, and $P_{pore}$ are the saturation pressure, minimum pressure, yield stress of pores, and the pore pressure.

Cavitation occurred when the values of NDCav>7, and no appearance of cavitation occurred when the values of NDCav<2. Researchers put forward an approach with two equations to calculate NDCav: when $T_{deso}$ is close to 0, NDCav is equivalent to $M_{y}$; when $T_{deso}>2$, the relationship between NDCav and the other two parameters can be approximately described as Equation (17).

(17) $${NDCav}^{\prime }=\frac{M_{y}}{2T_{deso}}$$

In their study, they mainly used NDCav to estimate the occurrence of cavitation with geometry, operation conditions, and material properties.

#### 5.2.3. Numerical Assessment of Maximum Decompression Rate

Based on the non-dimensional assessments of decompression damage mentioned above, Maximiliano et al. [74] proposed algebraic equations to evaluate the critical maximum decompression rate, $K_{safe}^{}$ (based on $K=\Delta P/t_{deso}\begin{array}{cc} , & \Delta P=P_{sat}-P_{\min} \end{array}$) that the specimens can endure before cavitation starts. 

The expression of $K_{safe}^{}$ for HDPE (shown as Equation (18)) was first proposed with the safe zone in the simulation results of pore pressure.

(18) $$K_{safe}^{HDPE}=\frac{0.32}{l^{2}}\begin{array}{cc} , & \frac{\Delta P}{P_{y}} \end{array}>2$$

Later, for the sake of a general law available to other materials, the authors extended their analyses to generalization of $K_{safe}^{}$ in both numerical (${K_{safe}}^{\prime }$) and mathematical (${K_{safe}}^{\prime \prime }$) approaches:

(19) $${K_{safe}}^{\prime }=\frac{\beta^{\prime }}{l^{\alpha }}\begin{array}{cc} , & \frac{\Delta P}{P_{y}} \end{array}>2$$

where α and $\beta^{\prime }$ are factors decided by the material’s diffusivity property D and mechanical property $\sigma_{y}$, and l is the half thickness. α is confirmed to be 2 with several simulations. $\beta^{\prime }$ is obtained as the fitting of β, which is a function of D and $\sigma_{y}$.

(20) $${K_{safe}}^{\prime \prime }=\left\{\begin{array}{l} \begin{array}{cc} \left(\frac{P_{y}\Delta P}{0.9\Delta P-0.8P_{y}}\right)\frac{D}{l^{2}} & 1<\frac{\Delta P}{P_{y}}<2 \end{array}\\ \begin{array}{cc} 2P_{y}\frac{D}{l^{2}} & \frac{\Delta P}{P_{y}}>2 \end{array} \end{array}\right.$$

Due to the absence of $\Delta P<2P_{y}$ limiting ${K_{safe}}^{\prime }$’s application,${K_{safe}}^{\prime \prime }$ was developed to complement the absent relationship between ΔP and $2P_{y}$. As the liner was safe from cavitation when $0<\Delta P/P_{y}<1$, the equation system of ${K_{safe}}^{\prime \prime }$ only considered two terms: $1<\Delta P/P_{y}<2$ and $\Delta P/P_{y}>2$.

## 6. Future Research Directions

The gas decompression damage to various polymers, particularly elastomers, has been studied for decades. However, failure studies of plastic materials suitable for the liner of type IV hydrogen tanks need to be comprehensive and mature. There is still much work that needs further study.

1. The liner design is of interest for damage minimization. Decompression damage is mainly determined by the diffusion and mechanical properties of the liner materials. Firstly, in the liner’s molding process, the molding method and parameters largely affect the material properties at the microscopic level. Molecular orientation is common in injection molding [93] and leads to the anisotropy of materials. The fusion temperature can influence the degree of crystallinity [94]. The effect of the molding process should be further investigated. Secondly, additives play a vital role in changing the properties of polymers and the nature of the damage. The introduction of novel additive strategies is a potential method to obtain desired properties and thereby minimize damage. Furthermore, Klopffer et al. [95] proposed innovative multilayer systems (applying a layer of Ethylene Vinyl Alcohol) and obtained excellent permeation resistance compared to monolayer systems. Therefore, the design of liner materials and systems could be a research interest to reduce the possibility and extent of damage.
2. The operational conditions will age the polymers and accelerate material degradation, making the liner more subject to damage. The characteristics of primary crystals of thermoplastics changed after long-term hydrogen exposure at various temperatures [96]. This is considered to be the effect of the annealing temperature. The crystallinity of PE was shown to increase under the high-pressure hydrogen conditions, but was restored to its initial value after decompression [97]. Considering the real practical application of the tanks, the effect of time and hydrogen exposure cycles on polymers’ properties may be prominent. Systematic and detailed research on the effect of operational conditions on material properties related to decompression damage is indispensable.
3. Water absorption should be emphasized when PA materials are chosen as the liner of type IV hydrogen storage tanks. It will directly affect the dimensional stability and the physical properties of the products [98]. Polyamide materials contain plenty of amide groups, making them sensitive to water from the environment (typically, the water absorption increases with the density of the amide group) [99]. The formation of hydrogen bonds between water molecules and the polar structure of PA decreases the density of hydrogen bonds between PA polymer chains. As a result, the intermolecular force decreases, and then the mobility of chains increases. It is reported that water absorption significantly impacted some properties of PA and its composites, such as thermal properties [100] and mechanical properties [101,102]. Randhawa et al. [100] utilized the SEM and XRD results of dry PA and water-treated PA to prove the generation of voids and the decrease in crystallinity after water immersion, which were responsible for the degradation of materials’ strength and hardness. In Ksouri et al.’s study, crazing appeared on the surface of long-term water-soaked PA6 and extended to the sample’s interior [103]. Water-induced voids and crazing may promote gas diffusion and weaken materials’ permeation resistance. Thus, the effect of ambient humidity on the decompression damage of polyamide materials cannot be ignored in future research.
4. The predictive model is a convenient numerical tool for predicting the decompression damage of materials at specific operational conditions. Some parameters used in current models are the function of temperature and pressure but are considered constants, such as solubility, diffusivity, and yield stress. Therefore, further work could improve the accuracy of parameters and the model complexity to present the damage morphology better. Enriching the database for polymers’ transportation and mechanical properties at various temperatures and pressures is crucial and meaningful work for predictive models.
5. The self-healing of polymers is gaining growing interest. Damaged materials can recover their original mechanical properties by self-healing. There are two main approaches to self-healing [104]: (1) intrinsic self-healing, repairing the damage by the chemical bonds inside the polymer itself, and (2) the extrinsic self-healing, which works using the release of healing agents. Blasizik et al. [105] believed that the potential for self-healing exists in thermosets, thermoplastics, and elastomers. Thermoplastics self-heal by joining the surfaces of cracks and then re-entangling polymer chains [106]. The application of self-healing to polymer liners will hinder the propagation of the damage and improve the service life of the type IV tank. Therefore, self-healing could be a future direction in the development of polymer liners for type IV tanks.

## 7. Conclusions

In the field of hydrogen energy, applying gaseous hydrogen storage tanks is paramount. The type IV hydrogen tank has gradually become the primary on-board compressed gas storage solution. Improving its high hydrogen storage capacity can satisfy the demand for a more extended driving range. The most significant difference between the type IV tank and the other three types of tanks is the liner material. The hydrogen gas can permeate through the polymer liner based on the solution–diffusion mechanism. When hydrogen is released rapidly at a high pressure, decompression damage may occur inside the liner, and the harsher the decompression conditions are, the more severe the damage becomes. Thus, guaranteeing polymer liners’ reliability and safety matters in the popularization and application of type IV hydrogen tanks. In order to study internal decompression damage, the first step is to characterize it. The characterization methods and quantitative evaluations for internal damage of polymers are summarized in this paper. The decompression damage depends on many factors. This paper then discusses affecting factors at the material level and operational conditions. These can guide researchers and manufacturers to improve the related material properties and explore rational system control strategies for gas release. Moreover, the internal decompression-damage-predictive models and assessments in lab-scale conditions are reviewed in the literature. These meaningful numerical tools are of great interest to avoid decompression damage at a lower cost. Finally, future research directions on decompression damage of polymer liners are proposed. The research findings will guide future design and application activities of type IV hydrogen storage tanks.

## Figures and Tables

**Table 1:** Evolution of mechanical properties after decompression damage.

Mechanical Properties	Evolution	References
Stiffness	↓	[28]
Yield stress	↓	[28]
Rupture stress	↓	[28]
Modulus	↓	[28,36]
Elongation at break	↑	[28]

Note: ”↑” represents an increase in mechanical properties, ”↓” represents a decrease in mechanical properties.

## Data Availability

Not applicable.
